# Prediction and comparison of postural discomfort based on MLP and quadratic regression

**DOI:** 10.1002/1348-9585.12292

**Published:** 2021-11-11

**Authors:** Jinwon Lee, Jaejin Hwang, Kyung‐Sun Lee

**Affiliations:** ^1^ School of Mechanical Engineering Korea University Seoul Republic of Korea; ^2^ Department of Industrial and Systems Engineering Northern Illinois University DeKalb Illinois USA; ^3^ Division of Energy Resources Engineering and Industrial Engineering Kangwon National University Chuncheon Republic of Korea

**Keywords:** deep learning, multilayer perception, postural discomfort, regression

## Abstract

**Objective:**

The objective of this study was to predict postural discomfort based on the deep learning‐based regression (multilayer perceptron [MLP] model).

**Methods:**

A total of 95 participants performed 45 different static postures as a combination of 3 neck angles, 5 trunk angles, and 3 knee angles and rated the whole‐body discomfort. Two different combinations of variables including model 1 (all variables: gender, height, weight, exercise, body segment angles) and model 2 (gender, body segment angles) were tested. The MLP regression and a conventional regression (quadratic regression) were both conducted, and the performance was compared.

**Results:**

In the overall regression analysis, the quadratic regression showed better performance than the MLP regression. For the postural discomfort group‐specific analysis, MLP regression showed greater performance than the quadratic regression especially in the high postural discomfort group. The MLP regression also showed better performance in predicting postural discomfort among individuals who had a variability of subjective rating among different postures compared to the quadratic regression. The deep learning for postural discomfort prediction would be useful for the efficient job risk assessment for various industries that involve prolonged static postures.

**Conclusions:**

The deep learning for postural discomfort prediction would be useful for the efficient job risk assessment for various industries that involve prolonged static postures. This information would be meaningful as basic research data to study in predicting psychophysical data in ergonomics.

## 1 INTRODUCTION

Work‐related Musculoskeletal Disorders (MSDs) have been an essential issue in numerous industrial fields. The negative impacts of MSDs have been well described, such as staff wellbeing, quality of life, job satisfaction, economic burden, sickness‐related absences and management procedures, and productivity at work.^1^ One of the most significant risk factors for developing MSDs is inappropriate or awkward postures.^2^, ^3^ Many jobs involve various awkward postures across different industrial sectors.^4^, ^5^ Awkward postures during work negatively impact various body joints. Previous studies have shown that awkward postures were associated with the increased musculoskeletal discomfort and strain of workers.^6^, ^7^, ^8^, ^9^

Musculoskeletal discomfort is often measured as a way to assess the MSDs in various work environments.^10^ Discomfort is related to grouped descriptors such as fatigue, pain, and strain in awkward postures. Discomfort could immediately reveal after performing postural activities that involve various joint positions.^11^ Many previous studies tended to focus on the whole‐body discomfort rather than body segment‐specific discomfort.^12^, ^13^ According to recent study results, the postural discomfort was greatly affected by the improper posture of a specific joint.^14^ Measurement of postural discomfort could also be useful by many researchers and practitioners because it does not require a biosensor or equipment.

Artificial neural networks have been broadly used to predict different body postures and discomfort had developed a prediction model of human reach posture based on psychophysical discomfort data using a regression model.^15^, ^16^ The study on postural discomfort prediction was mainly related to the car seat, car driver, and reach to grasp postures.^17^, ^18^, ^19^ In recent postural discomfort studies, they had developed a posture discomfort prediction model of awkward working postures in the manual assembly process.^20^ Hwang and Lee^14^ had evaluated the classification of whole‐body postural discomfort using cluster analysis.

In a recent study, Lee et al.^21^ compared the accuracies of squat posture classification using conventional machine learning and deep learning. They mentioned that the results obtained using deep learning were superior to those obtained using conventional machine learning. Yang et al.^22^ classified work‐related physical load levels in construction using deep learning models. Deep learning consisted of multiple neural layers for pattern classification or feature learning. Examples of deep learning models included a variety of structures, such as auto‐encoders, restricted Boltzmann machines, convolutional neural networks (CNNs), and recurrent neural networks (RNNs).^23^ Most studies have been using the deep learning algorithm to predict posture by converting it to three‐dimensional postures based on two‐dimensional image file data.^24^, ^25^, ^26^

Deep learning algorithms have been actively used to predict posture. Some studies have predicted discomfort based on a specific posture, but it was limited to sitting and driver postures. However, a lack of studies predicted the discomfort of various combined postures occurring in the workplace using deep learning algorithms. Therefore, the purpose of this study was to predict the degree of discomfort using a deep learning algorithm based on data on the discomfort levels of various combined postures that may occur in the workplace.

## 2 METHODS

### 2.1 Participants

A total of 95 participants (42 males and 53 females) were recruited for this study. The inclusion criteria consisted of: (1) participants did not have a present symptom of the MSD, and (2) participants had no previous history of the MSD. The average and standard deviation of age, height, and weight were 22.5 ± 2.8 years, 170.8 ± 6.5 cm, and 62.1 ± 8.1 kg, respectively.

### 2.2 Experimental protocol

A repeated‐measures laboratory study was conducted. A total of 45 different postural interactions were performed per participant. These postures were based on the interactions of 3 neck angles (0°, 20°, and 40°), 5 trunk angles (0°, 20°, 40°, 60°, and 80°), and 3 knee angles (0°, 30°, and 60°).^14^ The range of angles was determined according to the Rapid Entire Body Assessment (REBA).^27^ The order of 45 postural interactions was fully randomized to minimize the residual fatigue and systematic bias due to the order.

The research assistant used a goniometer to control each posture of the participant. For each assigned posture, the participant maintained it for 3 s, which was controlled by the beep sound.^14^ To minimize the fatigue, a 1‐min break was provided between tasks. Right after each posture, participants rated their overall discomfort based on the 10‐point Likert scale questionnaire. The lowest number indicated the least discomfort, and the highest number represented the greatest discomfort.

### 2.3 Data analysis

Several demographic variables (gender, weight, height) were collected to find variables that influence postural discomfort in addition to various neck angles, truck angles, and knee angles. Table 1 shows the categorical data and description of each variable. Height, weight, and exercise were expressed by categorization divided into certain sections. Model 1 and model 2 were constructed to verify the model performance according to the difference in variables. Model 1 included all variables (gender, weight, height, exercise, neck angle, trunk angle, knee angle). Model 2 covered the gender and the body segment angles (neck angle, trunk angle, knee angle).

**TABLE 1:** Categorical data and description of each variable

Categorical value	1	2	3	4	5	6	7	8
Gender	Male	Female						
Weight (kg)	≥40 and <50	≥50 and <60	≥60 and <70	≥70 and <80	≥80 and <90	≥90		
Height (cm)	≥145 and <150	≥150 and <155	≥155 and <160	≥160 and <165	≥165 and <170	≥170 and <175	≥175 and <180	≥180
Exercise	≥1 h per day	No						

To predict the discomfort based on different postural interactions of the neck, trunk, and knee and demographic variables, both polynomial regressions and deep learning‐based regression were considered as prediction models. For the polynomial regressions, quadratic polynomial regressions were used to consider nonlinear relationships between postures and discomfort. For the deep learning‐based regression, the Multilayer Perceptron (MLP) regression was used.

**FIGURE 1:**
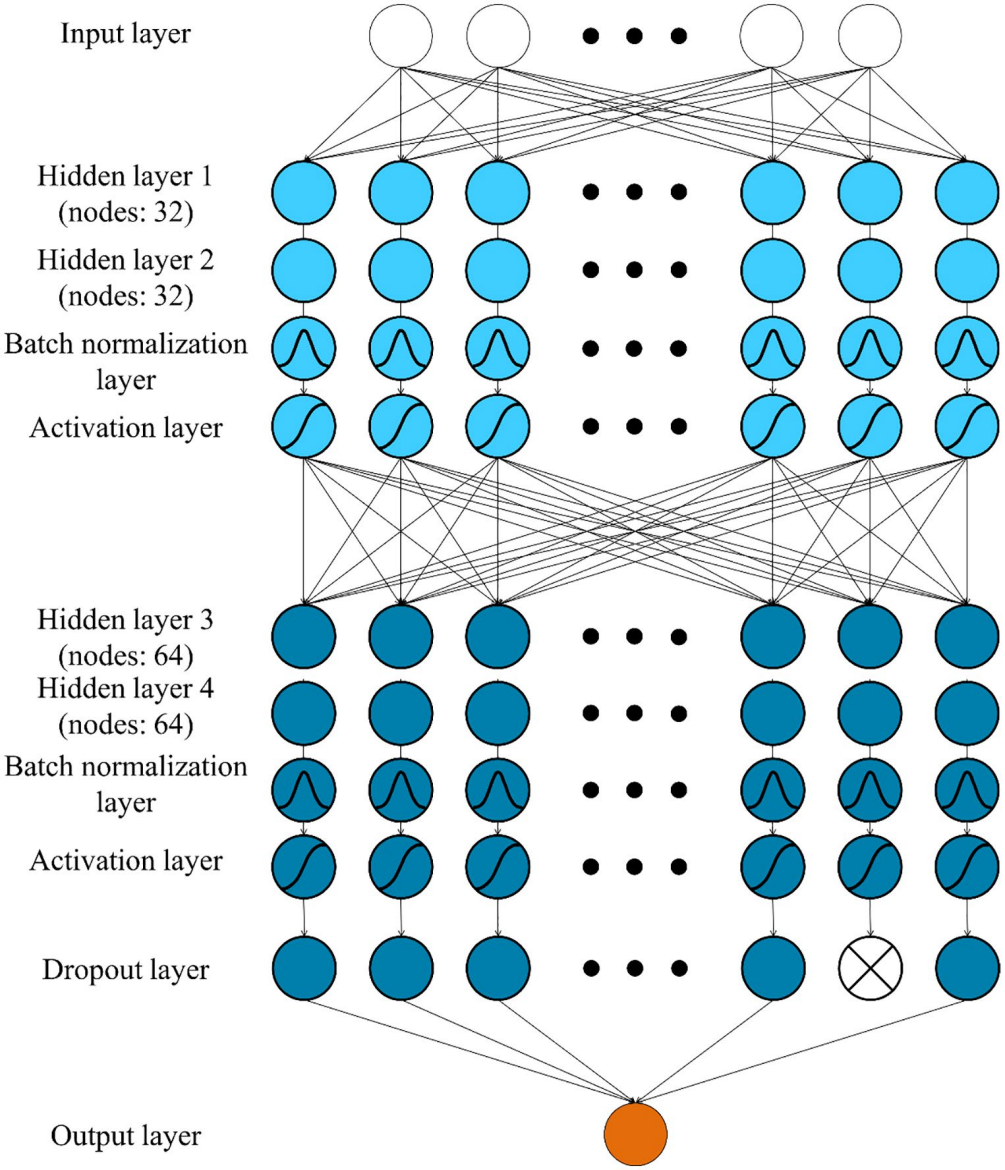
Multilayer perceptro network architecture

Figure 1 depicts a neural network architecture to predict postural discomfort. The networks consisted of the combination, including, input layer, hidden layer, batch normalization layer, activation layer, dropout layer, and output layer. The input layer depended on the number of input variables. Hidden layers had 32, 32, 64, 64 nodes, respectively. To make learning fast and stable, batch normalization was performed after the hidden layer. Activation layers utilized tanh function. The dropout layer blocked the signal going to the node of the next layer for robust training. The output layer resulted in postural discomfort.

The dataset of 95 participants was divided into 86 for training (90%) and 9 for testing (10%). Since postural discomfort varied greatly depending on the individual, the training dataset and the testing dataset were divided by considering the maximum, average, and minimum values of independent participants. In order to augment the training datasets, we added Gaussian noise to postural discomfort within a small variation range (*x* ± 0.05 × σ). In order to be less affected by the randomly segmented training dataset, k‐fold cross‐validation was employed with *K* = 4. The Adam optimizer Kingma and Ba^28^ was utilized with a learning rate of 0.001 for training. The batch size was 128, and all models were trained with the root mean square error (RMSE) as a loss function. To prevent overfitting, the model was trained for a maximum of 50 epochs. Our network was developed with python 3.7 and Keras 2.1.

In order to analyze the prediction performance by regression models, regression analysis and classification analysis methods were applied. MLP regression and quadratic regression models were trained with a training dataset (86 participants). Input data of the test dataset (nine participants) estimated the postural discomfort by the pre‐trained MLP regression and quadratic regression. For the performance measures to compare and validate two regression models, conventional regression analysis was employed—root mean squared error (RMSE), average & standard deviation, Pearson's correlation coefficient (*R*). Also, classification analysis was used to categorize various postural discomfort groups with clustering, confusion matrix, and scatter plot. To categorize varying postural discomfort groups, postural discomfort was classified into three levels: low, moderate, and high based on our previous study finding.^14^ In classification analysis, accuracy, recall, precision, and f1‐score were used as the evaluation metrics.^29^ Accuracy was the number of correctly predicted data divided by the total number of data (1). The recall was a measure of how many truly relevant results were returned and could be calculated by the measure of our model correctly identifying true positives (2). The precision was a measure of result relevancy and could be calculated as the ratio between the True Positives and all the Positives (3). F1 Score was the weighted average of precision and recall. Therefore, this score took both false positives and false negatives into account (4).

(1) $$\text{Accuracy}=\frac{\text{True}\;\text{positives}+\text{True}\;\text{Negatives}}{\text{All}\;\text{data}},$$

(2) $$\text{Recall}=\frac{\text{True}\;\text{positives}}{\text{True}\;\text{positives}+\text{False}\;\text{Negatives}},$$

(3) $$\text{Precision}=\frac{\text{True}\;\text{positives}}{\text{True}\;\text{positives}+\text{False}\;\text{Positives}},$$

(4) $$F1‐\text{Score}=\frac{2\times \text{Precision}\;\times \;\text{Recall}}{\text{Precision}\;+\;\text{Recall}}.$$

## 3 RESULTS

The overall mean and standard deviation of ground truth postural discomfort (reference) was 4.327 (±2.061). Since there was no big difference between the training curve and the validation curve, over‐, under‐fitting did not appear to have occurred.

A comparison of conventional regression analysis for Model 1 and Model 2 is shown in Table 2. The quadratic regression showed better performance than the MLP regardless of models 1 and 2. For the comparison between models 1 and 2, MLP showed an increased performance with Model 1 compared to Model 2. For the quadratic regression, there were no practical differences in the performance between models 1 and 2.

**TABLE 2:** Comparison of the performances between models with conventional regression analysis

Model type	Regression	AVG (STD)	RMSE	*R*
—	Ground truth	4.327 (2.061)	—	—
Model 1	MLP	4.853 (1.845)	1.922	0.555
	Quadratic	4.150 (1.385)	1.599	0.636
Model 2	MLP	5.334 (1.374)	1.902	0.622
	Quadratic	4.335 (1.350)	1.562	0.651

Table 3 describes the classification analysis for models 1 and 2. For the accuracy variable, there was no difference between MLP and quadratic regression within model 1, whereas quadratic regression showed higher accuracy than MLP within model 2. For the precision variable, MLP showed higher precision than quadratic regression within model 1, while there was no difference of precision within model 2. For the recall variable, quadratic regression showed higher recall performance than MLP within model 1, whereas there was an opposite pattern within model 2. For F1‐Score variable, MLP showed consistently higher values than quadratic regression across models 1 and 2.

**TABLE 3:** Comparison of the performances between models with classification analysis

Model type	Regression	Accuracy (%)	Precision (%)	Recall (%)	F1‐score (%)
Model 1	MLP	57.28	58.86	57.20	58.02
	Quadratic	57.03	54.14	59.65	56.76
Model 2	MLP	43.95	50.33	54.39	52.28
	Quadratic	57.53	51.11	43.89	47.21

**FIGURE 2:**
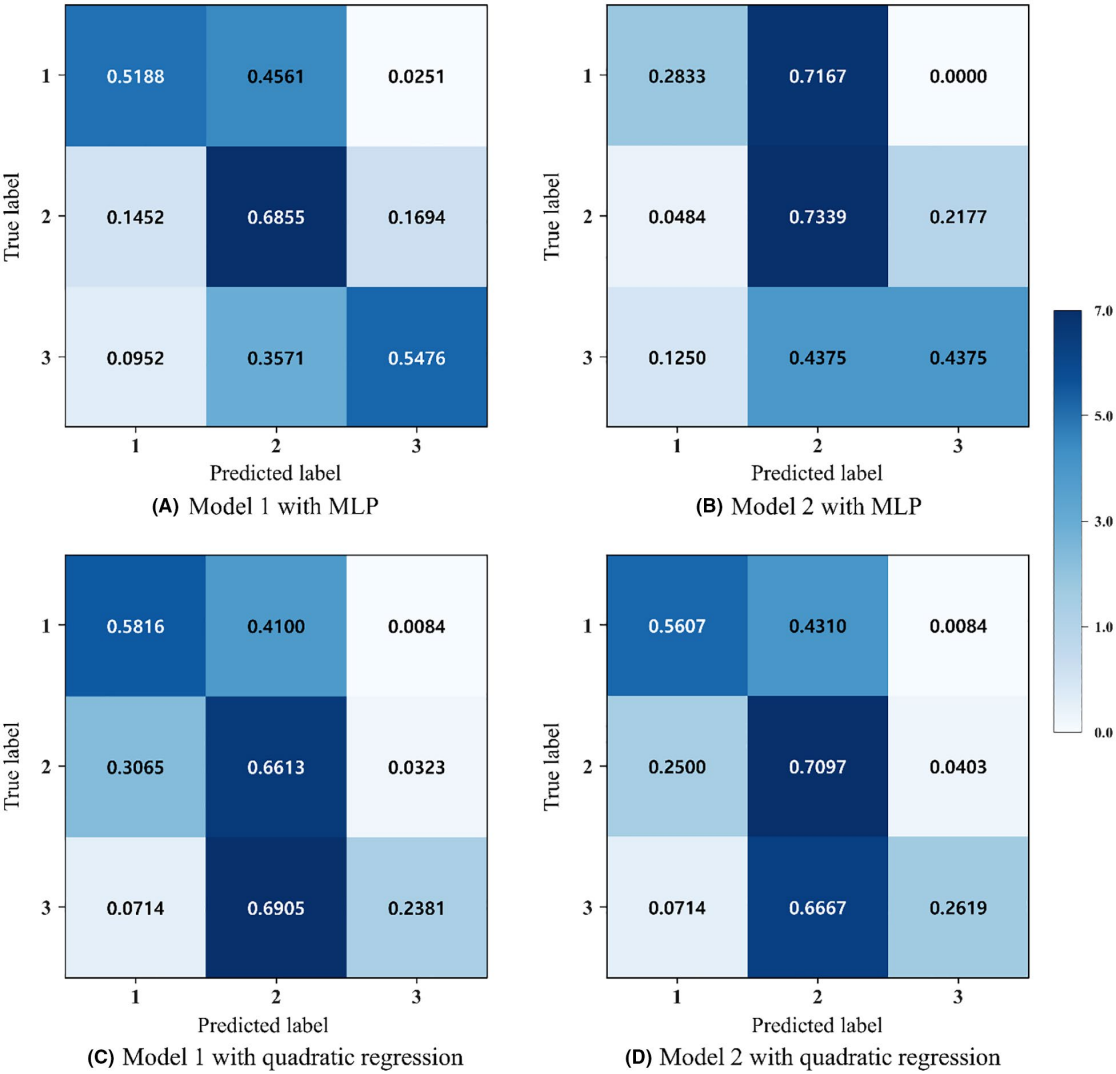
Confusion matrix for classification: (A) model 1 with MLP, (B) model 2 with MLP, (C) model 1 with quadratic regression, and (D) model 2 with quadratic regression

Figure 2 described the ground truth and predicted value results as a confusion matrix. The confusion matrix was close to the diagonal matrix illustrating the effectiveness and accuracy of the MLP regression.

**FIGURE 3:**
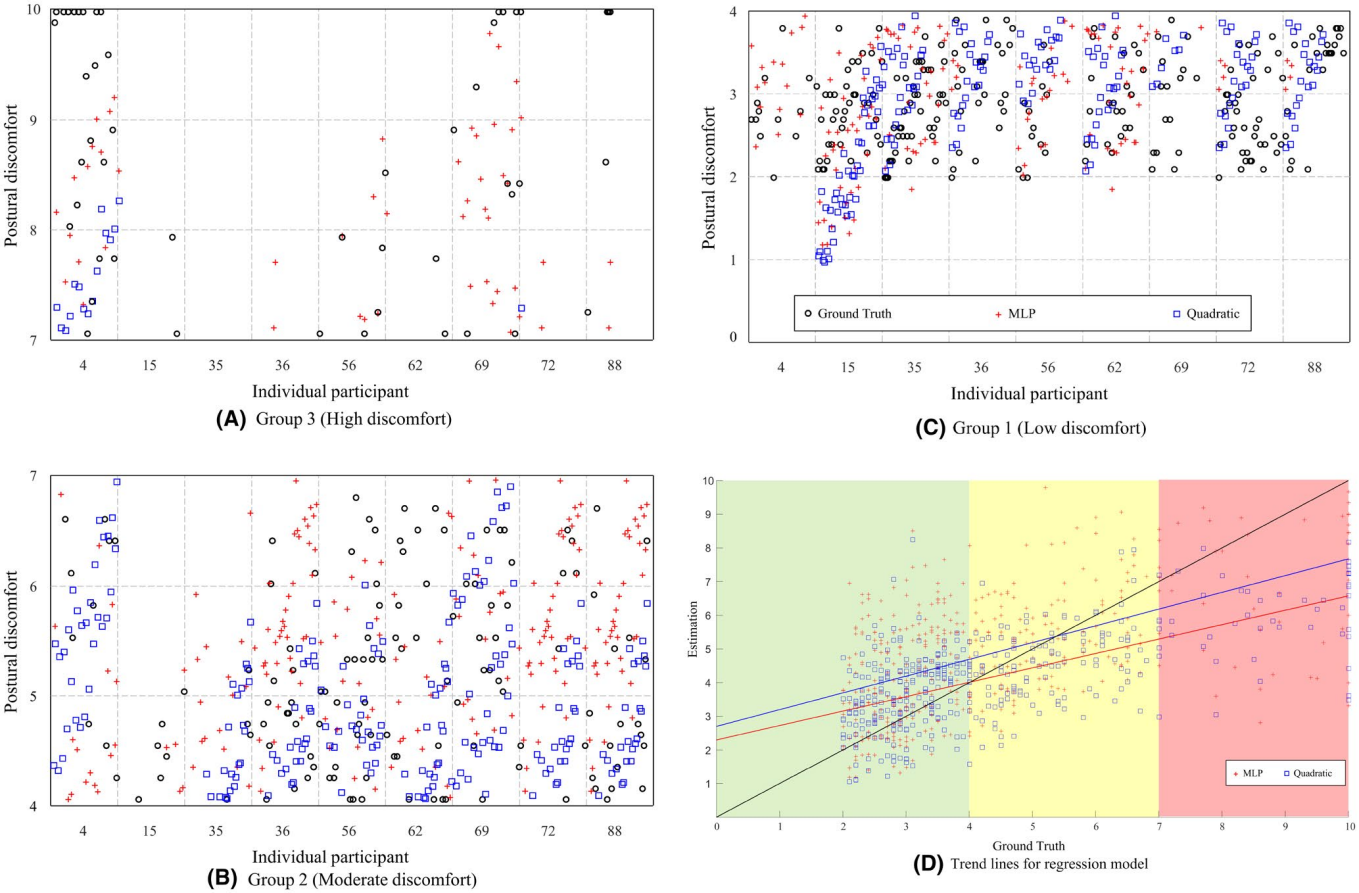
Predicted the postural discomfort by MLP and quadratic regressions for model 1: (A) group 3 (high discomfort), (B) group 2 (moderate discomfort), (C) group (low discomfort), and (D) trend lines for regression model. Black circle, red cross, blue rectangle indicates ground truth, MLP, quadratic regression, respectively

Figure 3 illustrates the individual value of the ground truth and predicted values by MLP and quadratic regression using model 1 for postural discomfort. For Figure 3A–C, the *X* axis indicates the different participant numbers, and the *Y* axis shows the postures discomfort, including the ground truth, predicted values from the MLP, and quadratic model. In group 3 (high discomfort), the number of data (portion) of the ground truth, MLP, and quadratic regressions were 42 (10.4%), 50 (12.3%), and 16 (4.0%), respectively. In group 2 (moderate discomfort), the ground truth, MLP, and quadratic regressions data sizes were 124 (30.6%), 209 (51.6%), and 209 (51.6%), respectively. In group 1 (low discomfort), the ground truth, MLP, and quadratic regressions data sizes were 239 (59.0%), 146 (36.0%), and 180 (44.4%), respectively. This difference was not substantial in moderate (Figure 3B) and low discomfort (Figure 3C) groups. As seen in Figure 3D, both MLP and quadratic models overestimated the postural discomfort in the low discomfort group, whereas they underestimated the values in the high discomfort group. For the high discomfort group, the MLP model showed less underestimation magnitude than the quadratic model.

## 4 DISCUSSION

In this paper, we predicted the postural discomfort using MLP regression and quadratic regression for model 1 (gender, height, weight, exercise, and body segment angles) and model 2 (gender and body segment angles). To compare the performance between MLP regression and quadratic regression for the postural discomfort, conventional regression analysis and classification analysis were employed. MLP and quadratic regression showed similar performance of the prediction in the moderate postural discomfort group, but MLP showed higher performance than quadratic regression in the high postural discomfort group. Especially, MLP of model 1 classified the high postural discomfort group with an accuracy of 54.76% compared to other prediction methods.

For the overall analysis, quadratic regression tended to show higher performance than MLP. This could be partially related to the distribution of the test dataset. Among the 405 test data, the high postural discomfort group (group 3) of test data was 42, which is only 10%. Also, the average and standard deviation of group 1 (2.960 ± 0.575) and group 2 (5.376 ± 0.878) were concentrated in between 3 and 5. Since quadratic regression finds the average value rather than the group 3 data, the performance is higher than that of MLP.

Although the quadratic regression showed better performance than MLP overall, there was a different result when analyzing the performance by specific postural discomfort groups. The MLP showed significantly improved performance (24% to 30% higher) than quadratic regression, especially in the high postural discomfort group (group 3). This was also supported by the confusion matrix in Figure 2. In the confusion matrix, the quadratic regression's performance was the highest in the moderate discomfort group (group 2), but there was a dramatic decline of the performance in the high discomfort group (group 3). This indicated that the quadratic regression's prediction was conservative and tended to predict values close to the average values. On the contrary, MLP showed more balanced performance across three postural discomfort groups. Especially with Model 1, MLP showed only slightly decreased performance in the high postural discomfort group (group 3) compared to the moderate postural discomfort group (group 2). Quadratic regression tended to predict values only within the moderate postural discomfort group regardless of the ground truth. MLP regression classified high postural discomfort values better than quadratic regression. This suggests that MLP would have an advantage in predicting postural discomfort especially in high postural discomfort levels. As shown in Figure 2, the MLP has less accuracy by 7% compared to quadratic regression in low discomfort. However, the MLP showed higher accuracy by 20% than quadratic regression in high discomfort. Especially, workplace injuries and health could be more strongly related to high discomfort postures. This indicates the importance of predicting high discomfort postures, as MLP outperformed the quadratic regressions. Since high postural discomfort values would be positively associated with the development of MSDs, the impact of the MLP approach would be expected to be substantial.

The different performance of individual participants between quadratic regression and MLP could also be assessed using scatter plots (Figure 3). When looking at the postural discomfort of the nine participants, some participants gave high scores such as #4 and #69, while others gave low scores such as #35, #36, and #72. High variability of postural discomfort across participants could be related to an individual's perception, sensibility, and physical conditions. Quadratic regression did not capture the variance between individual participants well since their prediction was conservative. As a result, quadratic regression showed a low performance of predicting the postural discomfort of individuals who tended to rate high postural discomfort values and have high variability of postural discomfort among 45 postural conditions. Unlike quadratic regression, MLP showed good prediction results for participants #4 and #69 in the high postural discomfort group (Figure 3A). As shown in high discomfort group (Figure 3A), MLP predicted more similar to the ground truth compared to quadratic regression, especially for participants of #4, #56, #69, #88. In addition, for the low postural discomfort group (Figure 3C), MLP made accurate predictions for #15 participants who gave low overall postural discomfort scores. This suggests that MLP would have the potential to learn and predict the variability of participant's subjective postural discomfort among different combinations of body postures. This was in line with our previous study showing that MLP showed higher performance in predicting the grip strength compared to linear, quadratic, and cubic regressions, especially for individuals who had high variability of grip strength across conditions.^30^ This supports the MLP’s strength in solving classification and regression problems identified from previous studies.^31^

We predicted subjective postural discomfort by demographic information of participants and body segment angles (neck, trunk, knee) using MLP regression. This prediction of various postural discomfort levels will be useful in various industries that involve prolonged static postures such as cashiers, surgeons, and dentists. The predictive model developed in this study could be useful to be embedded in the video‐based postural assessment tool. Our findings of predicting postural discomfort could advance our fundamental understanding of estimating subjective measurements commonly used in the ergonomics field.

There were several limitations in this study. First, the dataset was built with 95 subjects. Although the present study showed the meaningful results based on this small sample size, further experiments with a larger sample size could yield higher prediction performance. Second, this study did not consider additional properties such as the age of participants and underlying disease that may affect postural discomfort. Since we only recruited university students, we could not train postural discomfort of different age levels of participants. Since age is known as a critical factor affecting the postural discomfort, age‐specific prediction of postural discomfort could be studied in the future.^32^ Lastly, despite of promising results of MLP regression in high postural discomfort groups, the overall prediction performance was deemed low (accuracy is about 57%). The performance could be further improved by adjusting the model structure or utilizing different deep learning algorithms.

## 5 CONCLUSION

This study applied the MLP regression and quadratic regression to predict postural discomfort and compared the performance of prediction using conventional regression analysis and classification analysis. Quadratic regression of model 1 (including all variables) revealed a good prediction performance in the conventional regression analysis, and MLP of model 1 showed a great performance in classification analysis. Especially, MLP of model 1 showed a higher accuracy of 54.7% than the other methods (24%~30%), especially in the high postural discomfort group. This information would be meaningful as basic research data to study in predicting psychophysical data in ergonomics.

## CONFLICT OF INTEREST

The authors declare that there is no conflict of interest.

## DISCLOSURE

*Ethical approval*: The research design in this study was reviewed and passed by the Human Subject Committee. *Informed consent*: all the respondents signed the Human Subject Consent. *Animal Studies*: N/A.

## AUTHOR CONTRIBUTIONS

Lee K‐S conceived the ideas. Hwang JJ contributed to literature review. Lee K‐S collected the data. Lee JW analyzed the data. Lee K‐S was involved in research design, literature discussion, and data analysis. Lee K‐S, Hwan JJ, and Lee JW led the writing.

## Data Availability

The data that support the findings of this study are available on request from the corresponding author. The data are not publicly available due to privacy or ethical restrictions.

## References

[1] Long MH , Bogossian FE , Johnston V . Functional consequences of work‐related spinal musculoskeletal symptoms in a cohort of Australian midwives. Women Birth. 2013;26:e50‐e58. 23098669 10.1016/j.wombi.2012.09.005

[2] Alavinia SM , van Duivenbooden C , Burdorf A . Influence of work‐related factors and individual characteristics on work ability among Dutch construction workers. Scand J Work Environ Health. 2007;33(5):351‐357. 17973061 10.5271/sjweh.1151

[3] Monjezi N . Ergonomic Evaluation Posture of Sugarcane Workers using REBA Method. J Agric Mach. 2021;11:477‐489.

[4] Keyserling WM , Brouwer M , Silverstein BA . A checklist for evaluating ergonomic risk factors resulting from awkward postures of the legs, trunk and neck. Int J Ind Ergon. 1992;9:283‐301.

[5] Vandergrift JL , Gold JE , Hanlon A , Punnett L . Physical and psychosocial ergonomic risk factors for low back pain in automobile manufacturing workers. Occup Environ Med. 2012;69:29‐34. 21586759 10.1136/oem.2010.061770

[6] Chung MK , Lee I , Kee D . Assessment of postural load for lower limb postures based on perceived discomfort. Int J Ind Ergon. 2003;31:17‐32.

[7] Hwang J , Kong Y‐K , Jung M‐C . Posture evaluations of tethering and loose‐housing systems in dairy farms. Appl Ergon. 2010;42:1‐8. 20427034 10.1016/j.apergo.2010.03.008

[8] Lim C‐M , Jung M‐C , Kong Y‐K . Evaluation of upper‐limb body postures based on the effects of back and shoulder flexion angles on subjective discomfort ratings, heart rates and muscle activities. Ergonomics. 2011;54:849‐857. 21943119 10.1080/00140139.2011.600777

[9] Wang H , Hwang J , Lee K‐S , Kwag J‐S , Jang J‐S , Jung M‐C . Upper body and finger posture evaluations at an electric iron assembly plant. Hum Factors Ergon Manuf Serv Ind. 2014;24:161‐171.

[10] Marras WS . The future of research in understanding and controlling work‐related low back disorders. Ergonomics. 2005;48:464‐477. 16040520 10.1080/00140130400029175

[11] Reid CR , Bush PM , Karwowski W , Durrani SK . Occupational postural activity and lower extremity discomfort: a review. Int J Ind Ergon. 2010;40:247‐256.

[12] Genaidy AM , Karwowski W . The effects of neutral posture deviations on perceived joint discomfort ratings in sitting and standing postures. Ergonomics. 1993;36:785‐792. 8339718 10.1080/00140139308967942

[13] Kee D , Karwowski W . Ranking systems for evaluation of joint and joint motion stressfulness based on perceived discomforts. Appl Ergon. 2003;34:167‐176. 12628574 10.1016/S0003-6870(02)00141-2

[14] Hwang J , Lee K‐S . Classification of whole‐body postural discomfort using cluster analysis. Int J Environ Res Public Health. 2020;17:8314. 10.3390/ijerph17228314 PMC7697103 33182760

[15] Jung ES , Choe J . Human reach posture prediction based on psychophysical discomfort. Int J Ind Ergon. 1996;18:173‐179.

[16] Zhang B , Horváth I , Molenbroek JF , Snijders C . Using artificial neural networks for human body posture prediction. Int J Ind Ergon. 2010;40:414‐424.

[17] Kruizinga CP , Delleman NJ , Schellekens JM . Prediction of musculoskeletal discomfort in a pick and place task (a pilot study). Int J Occup Saf Ergon. 1998;4:271‐286. 10602622 10.1080/10803548.1998.11076394

[18] Olendorf MR , Drury CG . Postural discomfort and perceived exertion in standardized box‐holding postures. Ergonomics. 2001;44:1341‐1367. 11936827 10.1080/00140130110085358

[19] Porter JM , Gyi DE , Tait HA . Interface pressure data and the prediction of driver discomfort in road trials. Appl Ergon. 2003;34:207‐214. 12737920 10.1016/S0003-6870(03)00009-7

[20] Yonga T , Kanakana‐Katumba G , Mpofu K , Monzambe G . Prediction of postural discomfort impact on manual assembly: a workshop case study. Procedia Manuf. 2020;43:583‐589.

[21] Jaehyun L , Joo H , Junglyeon L , Chee Y . Automatic classification of squat posture using inertial sensors: deep learning approach. Sensors. 2020;20(2):361. 10.3390/s20020361 PMC7014149 31936407

[22] Yang K , Ahn CR , Kim H . Deep learning‐based classification of work‐related physical load levels in construction. Adv Eng Inform. 2020;45:101‐104.

[23] Biswas D , Cranny A , Gupta N , et al. Recognizing upper limb movements with wrist worn inertial sensors using k‐means clustering classification. Hum Mov Sci. 2015;40:59‐76. 25528632 10.1016/j.humov.2014.11.013

[24] Abobakr A , Nahavandi D , Hossny M , et al. RGB‐D ergonomic assessment system of adopted working postures. Appl Ergon. 2019;80:75‐88. 31280813 10.1016/j.apergo.2019.05.004

[25] Tang A , Lu K , Wang Y , Huang J , Li H . A real‐time hand posture recognition system using deep neural networks. ACM Trans Intell Syst Technol TIST. 2015;6:1‐23.

[26] Zhang H , Yan X , Li H . Ergonomic posture recognition using 3D view‐invariant features from single ordinary camera. Autom Constr. 2018;94:1‐10.

[27] Hignett S , McAtamney L . Rapid entire body assessment (REBA). Appl Ergon. 2000;31:201‐205. 10711982 10.1016/s0003-6870(99)00039-3

[28] Kingma DP , Adam BJ . A method for stochastic optimization. ArXiv Prepr. 2014;ArXiv14126980.

[29] Kim H , Yeo C , Cha M , Mun D . A method of generating depth images for view‐based shape retrieval of 3D CAD models from partial point clouds. Multimed Tools Appl. 2021;80:10859‐10880.

[30] Hwang JJ , Lee JW , Lee K‐S . A deep learning‐based method for grip strength prediction: comparison of multilayer perceptron and polynomial regression approaches. PLoS One. 2021;16(2):e0246870. 33571318 10.1371/journal.pone.0246870 PMC7877597

[31] Yilmaz L , Oquz K . Multiple regression, ANN (RBF, MLP) and ANFIS models for prediction of swell potential of clayey soils. Expert Syst Appl. 2021;80:10859‐10880.

[32] Gruevski KM , Callaghan JP . The effect of age on in‐vivo spine stiffness, postures and discomfort responses during prolonged sitting exposures. Ergonomics. 2019;62:917‐927. 30889363 10.1080/00140139.2019.1596317

